# Smoking dependence and anxio-depressive disorders in Tunisian smokers attending the smoking cessation clinic in a university hospital

**DOI:** 10.1186/s42506-019-0012-y

**Published:** 2019-02-20

**Authors:** Héla Ghali, Oussama Ben Rejeb, Sihem Ben Fredj, Salwa Khéfacha, Lamine Dhidah, Mohamed Ben Rejeb, Houyem Said Latiri

**Affiliations:** 1 Department of Prevention and Security of Care, Hospital of Sahloul, Faculty of Medicine of Sousse, University of Sousse, Sousse, Tunisia; 2 Department of Cardiology, Hospital of Farhat Hached, Faculty of Medicine of Sousse, University of Sousse, Sousse, Tunisia

**Keywords:** Smoking cessation, Anxiety, Depression, Anxio-depressive disorders

## Abstract

**Background:**

Smokers with comorbid anxio-depressive disorders are more prone to progress to a more severe level of dependence and to experience more severe nicotine withdrawal symptoms than smokers without anxio-depressive disorders.

**Aim of the study:**

To determine the relationship between tobacco dependence and anxio-depressive disorders as well as assessing their impact on the withdrawal.

**Methods:**

We conducted a cross-sectional study among attendees of the smoking cessation clinic of Sahloul University Hospital, *Sousse*, *Tunisia*, from December 2009 to May 2015. The monitoring of the attendees was performed through retrieving the records until May 2016 in order to verify their smoking cessation status at 1 year.

**Results:**

Overall, 534 smokers were included. We identified 315 smokers (59%) presenting an anxio-depressive disorder. Based on the HAD scale, we found 231 patients (43.4%) with anxiety disorders, 200 (37.6%) patients with depressive disorders, and 116 (21.8%) patients with anxio-depressive disorder.

In multivariate analysis, only a high number of consultation was associated with a better rate of tobacco cessation at 6 months. However, no factor was found linked to the relapse at 1 year.

**Conclusion:**

According to our results, only a high number of consultation was revealed as an independent factor of withdrawal for anxio-depressed smokers. It is necessary to simultaneously use the nicotinic substitutions and anxio-depressive treatment to ensure the tobacco cessation.

## Introduction

Smoking is now considered a chronic disease caused by nicotine dependence and is one of the main risk factors for various diseases [1]. By 2020, annual tobacco-related deaths are projected to increase to 7.5 million, accounting for 10% of all deaths in that year. Smoking is estimated to cause about 71% of all lung cancer deaths, 42% of chronic respiratory disease, and nearly 10% of cardiovascular disease. Smoking is also an important risk factor for communicable diseases such as tuberculosis and lower respiratory infections [2]. Around 80% of the 1.1 billion smokers worldwide live in low- and middle-income countries, where the burden of tobacco-related illness and death is heaviest [3].

Cigarette smoking behavior is defined as actively smoking one or more manufactured or hand-rolled cigarettes with the intention to inhale the tobacco smoke [4]. Smoking is also a social behavior that results in a physical and psychological dependence requiring a specific treatment. The CDC 2011 indicated that70% of smokers would like to quit smoking, and 50% report attempting to quit within the past year [5]. Nicotine dependence is the major obstacle to the success of quitting [6].

Despite the general advances in smoking control, the high rates of treatment failure in smoking cessation programs constitute a cause for concern. [7] Among the various complicating factors are high levels of anxiety, depression, and stress, as well as a low level of motivation for change, on the part of patients who seek treatment via smoking cessation programs [7–9].

There is now broad-based recognition employing representative and clinical samples that smokers are more likely to have a psychiatric disorder than non-smokers, and individuals with a psychiatric disorder are significantly more likely to smoke compared to persons without a psychiatric disorder [10]. Among the various psychiatric disorders implicated in smoking, depressive, and anxiety syndromes (i.e., emotional disorders) are particularly important to study because they are highly prevalent in the general population and remarkably comorbid with smoking [11]. There is also robust empirical evidence that elevated depressive and anxiety symptoms and emotional disorders increase the risk of smoking experimentation [12], progression to daily smoking [13], and development of nicotine dependence [14] and contribute to maladaptive cognitive–emotional reactions to tobacco [15].

Smoking incidence and maintenance generalizes across various emotional disorders, including major depression [16], dysthymia and minor depression [17], posttraumatic stress disorder [18], and other anxiety disorders including panic disorder, social anxiety disorder, and generalized anxiety disorder [19]. A systematic tracking of these disorders in smoking cessation clinic is necessary. Our study is attached in this context aiming to:

- Measure the frequency of anxio-depressive disorders among smokers of our smoking cessation clinic
- Assess the relationship between nicotine dependence and the severity of anxiety and depression among the smokers
- Assess the potential effect of these disorders on the tobacco cessation

## Participants and methods

### Type and population of the study

This was a cross-sectional study conducted in the smoking cessation clinic in the University Hospital of Sahloul in Sousse, Tunisia. We included in this study the records of all smokers who attended the clinic since the date of its creation in December 2009 until May 2015 (*n* = 534). The monitoring of the smokers was performed by retrieving the records until May 2016 in order to assess their smoking cessation status at 1 year. Some of these records had missing data about the status of smoking. Smokers with incomplete records were contacted in May 2016 by phone to give information on their status. Our clinic activities have begun in December 2009, and all the departments of the hospital were informed of its creation. This preventive clinic is linked to the Department of Prevention and Security of Care. It is open free of charge to the public including hospital staff, hospital patients, and community. It is provided by a team of two physicians specialized in counseling of smokers, two nurses, and a nutritionist when necessary.

### Data collection

Patients’ data were collected using their records of the smoking cessation clinic. These data included:

- Sociodemographic variables: age, sex, marital, and employment status, educational level, personal history of health problems, and lifestyle habits
- Characteristics of the smoking history: the duration of smoking, number of daily smoked cigarettes, the age of smoking initiation, and previous quit attempts
- Fagerstrom Nicotine Dependence Test (FTND) [20] was used to measure the level of nicotine dependence for smokers. FTND consists of six items and scoring for each item may differ from question to question. The cut-off score for FTND scale would be low dependence (0–4), moderate dependence (5–6), and high dependence (7–10).
- The exhaled carbon monoxide in air (CO) at the first visit [21]
- The perceived motivation to quit: assessed by the Lagrue and Legeron test [22]: smokers having a score equal or above 12 were considered as motivated to quit
- The score of perceived self-confidence to quit smoking: assessed by a Likert scale ranging from 0 (not confident at all) to 10 (extremely confident). Smokers having a score ≥ 5 were considered confident to quit.

To measure depression and anxiety, severity was evaluated on the basis of the Hospital Anxiety Depression Scale (HAD) [23]. Scores of 12 and 8 were respectively considered the threshold for anxiety and depression.

Other addictive behaviors were assessed by the interrogation: evaluation of the consumption of alcohol based on the number of glasses.

### The evaluation of the tobacco cessation

The smoking cessation was confirmed by the smoker himself and verified by the measurement of the CO in the expired air.

All patients were contacted and interviewed by telephone, which has allowed us to retrieve the information from the patients who have interrupted the follow-up. Non-responders at the date of the scheduled visit were considered as persistent smokers in the analysis of results.

In our study, successful smoking cessation defined as a complete and continuous abstinence was assessed at 1 week, at 1 month, at 3 months, at 6 months, and at 12 months. An exploratory study of the factors of successful smoking cessation was done in subjects who stopped smoking in about a week and maintained for 1 year or more among smokers who consulted more than twice.

### Statistical analysis

The statistical package SPSS 10.0 for Windows (IBM, USA) was used for the analyses. We compared anxiety and depression scores between patients abstinent and patients still smokers. Data were presented with frequencies, means, and standard deviations. Categorical variables were compared using the chi-square statistical method. Odds ratios and 95% confidence intervals were calculated. Differences between means for univariate analyses on continuously distributed variables were compared using Student’s *t* test or ANOVA test. Univariate then multivariate analyses were performed in order to identify the determinants of a successful smoking cessation among smokers with anxio-depressive disorders. For all statistical analyses, differences were considered significant at *p* value < 0.05.

## Results

### Population characteristics

During the study period, a total of 534 smokers were included. The participants were predominantly males (*n* = 500, 93.6%). Their mean age was 42.1 ± 13.8 years old with extremes ranging from 14 to 82 years old. The two age groups most represented were those of 45–54 years old (*n* = 139; 26%) and 25–34 years old (*n* = 129; 24.2%). Almost half of our population (49%) were heavy smokers (more than 20 cigarettes per day) and 48.9%of smokers were strongly addicted to nicotine (Fagerström score ≥ 7) (Table 1).

**Table 1:** Characteristics of smokers attending the smoking cessation clinic of the University Hospital Sahloul, Sousse, 2009–2015

Characteristic	Number (*n*)	Percentage (%)
Gender (*n* = 534)		
Male	500	93.6
Female	34	6.4
Age		
≤ 24 years	62	11.6
25–34 years	129	24.2
35–44 years	99	18.5
45–54 years	139	26
55–64 years	83	15.5
≥ 65years	22	4.1
Age (mean ± standard deviation)	42.1 ± 13.8 years	
Educational level (*n* = 534)		
Primary	158	29.6
Secondary	199	37.3
University	177	33.1
Employment status (*n* = 534)		
Student	41	7.7
Employee	400	74.9
Unemployed	93	17.4
Practice of at least 30 min of physical activity per week (*n* = 528#)	246	46.6
Alcohol intake (*n* = 466)	89	16.7
Medical history of high blood pressure (*n* = 534)	43	8.1
Medical history of diabetes mellitus (*n* = 534)	60	11.2
Medical history of coronary heart disease (*n* = 534)	32	6
Medical history of respiratory disease (*n* = 534)	32	6.2
Medical history of gastric pathologies (*n* = 534)	45	8.4
Age of smoking onset (*n* = 528#)		
≤ 12years	48	9
> 12 years	480	91
Number of daily smoked cigarettes (*n* = 534)		
< 20	272	51
≥ 20	262	49
Fagerström score (*n* = 534)		
< 7	273	51.1
≥ 7	261	48.9
Previous attempt to quit (*n* = 534)	281	52.6
Exhaled CO in air (*n* = 449#) (mean ± standard deviation)	14.86 ± 9.67	
Score of motivation (*n* = 534)		
≤ 12	147	27.5
> 12	387	72.5
Score of self-confidence to quit (*n* = 534)		
≥ 5	60	11.2
< 5	474	88.8

# Missing information

**Fig. 1:**
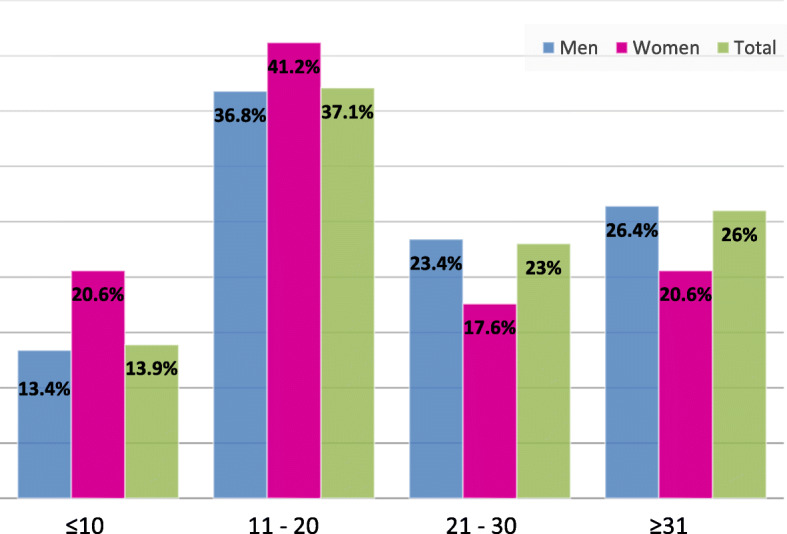
Distribution of the number of cigarettes smoked per day by smokers attending the smoking cessation clinic of the University Hospital Sahloul, Sousse, 2009–2015

The mean number of daily smoked cigarettes was 26.97 ± 14.6 cigarettes with extremes ranging from 1 to 100 cigarettes (Fig. 1).

The average score of nicotine dependence was 6.26 ± 2.38 with extremes ranging from 0 to 10.

**Fig. 2:**
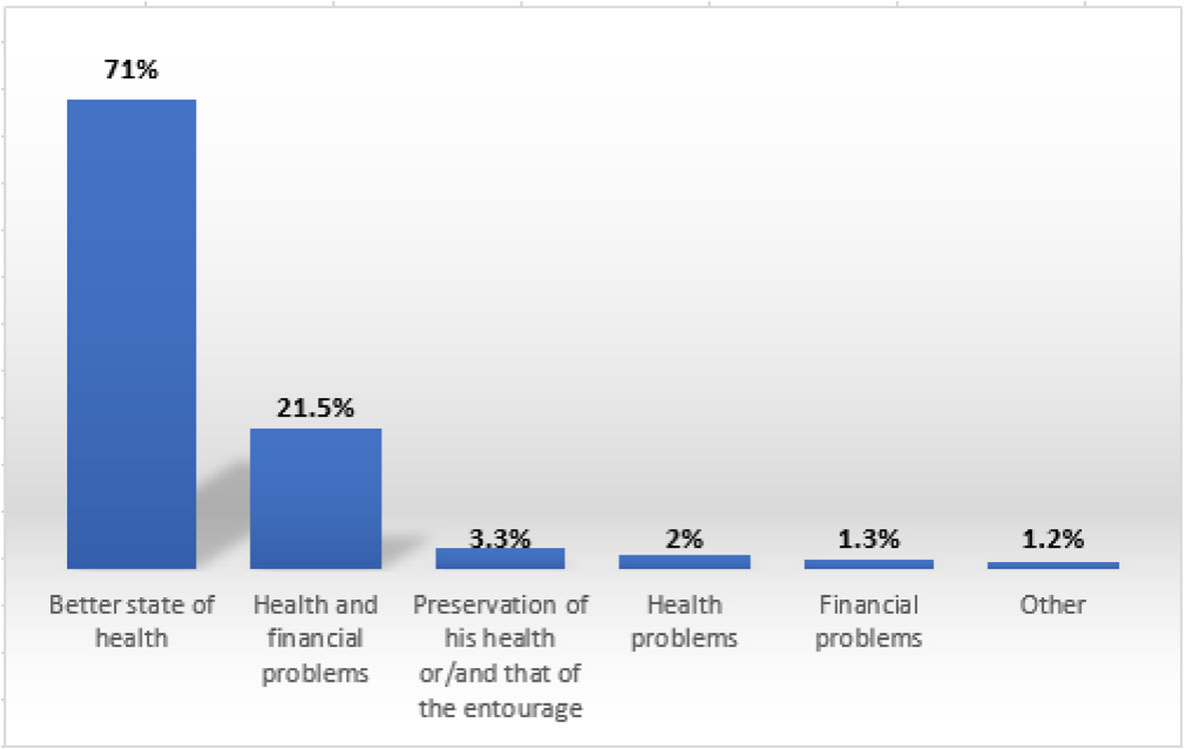
Distribution of smokers according to the reasons for the decision to quit smoking

72.5% of smokers were motivated to tobacco cessation. The average score of motivation was 14.36 ± 4.63. The search for a better state of health was the main reason inciting 71% of them to quit smoking (Fig. 2).

The measurement of the exhaled carbon monoxide in air (CO) was performed among 449 smokers. The average rate of exhaled CO in air was 14.86 ± 9.67 ppm (ppm) with extremes ranging from 1 to 60.

The consumption of alcohol was found in 16.7% of smokers. A cardiovascular history was reported in 20.9% in which the most frequent were the high blood pressure (*n* = 43; 8.1%) and coronary artery disease (*n* = 32; 6%). A medical history of respiratory disease was noted in 32 smokers (6.2% of cases). A history of depression has been found in 12 smokers (Table 2).

**Table 2:** Description of medical characteristics of smokers attending the smoking cessation clinic of the University Hospital Sahloul, Sousse, 2009–2015

Medical history	Number (*n*)	Percentage (%)
Cardiovascular history	110	20.9
Coronary artery disease	32	6
Obliterative arterial disease of the lower limbs	10	1.9
Stroke	6	1.2
Hypertension	43	8.1
Thrombophlebitis	12	2.3
Disorder of the Pace	7	1.4
Respiratory history	32	6.2
Chronic obstructive pulmonary disease	6	1.1
Bronchitis repetition	16	3.1
Lung emphysema	1	0.2
Asthma	6	1.2
Pulmonary tuberculosis	3	0.6
History of depression	12	2.3
Diabetes	60	11.2
Digestive history	45	8.4

### Anxio-depressive disorders and smoking cessation

**Fig. 3:**
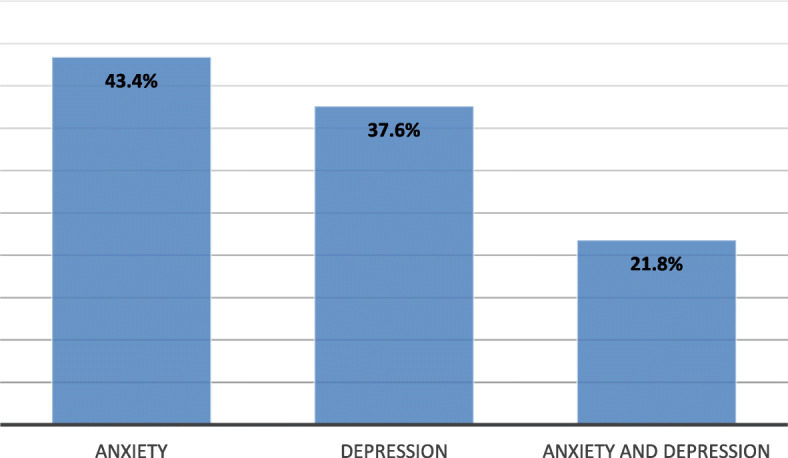
Frequency of anxio-depressive disorders at the time of the first consultation (the total does not sum up to exactly 100% because of multiple diagnoses)

We identified 315 smokers (59%) presenting an anxio-depressive disorder among the 534 attendees of the smoking cessation clinic. Based on the HAD scale, we found 231 patients (43.4%) with anxiety disorders (A-HAD scale ≥ 12), 200 (37.6%) with depressive disorders (D-HAD scale ≥ 8), and 116 (21.8%) patients with both anxiety and depression (Fig. 3).

The average score of anxiety was 10.67 ± 4.63 and that of depression was 6.49 ± 3.58.

A strong dependency was more common among anxious smokers (46.7%), followed by depressed smokers (43%), and the least was among the anxio-depressed smokers (one quarter). The average score of physical dependence was higher among smokers with an anxio-depressive disorder than among those who are not (6.67 ± 2.35 vs 5.67 ± 2.35) (Table 3).

**Table 3:** Anxio-depressive disorders according to Fagerström score among the attendees of the smoking cessation clinic of the University Hospital Sahloul, Sousse, 2009–2015

Psychological disorder	Percentages (%)	Fagerström score	*p*
Anxiety			
Yes (*n* = 231)	43.4	6.74 ± 2.36	< 10^−3^
No (*n* = 301)	56.6	5.90 ± 2.34	
Depression			
Yes (*n* = 200)	37.6	6.74 ± 2.43	< 10^−3^
No (*n* = 332)	62.4	5.98 ± 2.31	
Anxio-depressive disorder			
Yes (*n* = 116)	21.8	6.67 ± 2.35	0.001
No (*n* = 416)	78.2	5.67 ± 2.35	

Anxio-depressed smokers were less likely to experience successful tobacco cessation than non-anxio-depressed ones at 1 week, 1 month, 3 months, 6 months, and at 1 year. These differences were significant only at 1 month (Table 4). We have taken into consideration for the analyses only smokers who had more than two visits to the smoking cessation clinic.

**Table 4:** Distribution of smokers according to the time of tobacco cessation

	Tobacco cessation at				
	1 week (%)	1 month (%)	3 months (%)	6 months (%)	1 year (%)
Not anxio-depressive smokers (*n* = 217)	35.9	69.6	39.3	30.4	25
Anxio-depressed smokers (*n* = 315)	32.7	51.2	34.1	23.2	18.3
*p* value	0.43	< 0.001	0.21	0.06	0.07
Total (*n* = 534)	33.9	58.7	36.2	26.1	21

In the univariate analysis, advanced age and a high number of visits to the clinic were significantly associated with successful cessation at 6 months (Table 5). In the multivariate analysis, only a high number of visits to the clinic was an independent factor associated with smoking cessation at 6 months (*p* = 0.012; OR = 1.23; CI = [1.04–1.46]). The univariate as well as the multivariate analyses showed no factor significantly associated with the relapse at 12 months.

**Table 5:** Factors associated with a successful smoking cessation at 6 months among smokers with anxio-depressive disorders attending the smoking cessation clinic of the University Hospital Sahloul, Sousse, 2009–2015

	Smoking cessation at 6 months		OR [95% CI]	*p*
	Yes *n* (%)	No *n* (%)		
Gender			0.66 [0.15–2.87]	NS
Female	3 (30)	7 (70)		
Male	16 (22.2)	56 (77.8)		
Age (mean ± standard deviation)	48.05 ± 10.49	41.31 ± 13.27	1.04 [1–1.09]	0.05
Previous attempt to quit			1.08 [0.36–3.25]	NS
Yes	13 (23.6)	42 (76.4)		
No	6 (22.2)	21 (77.8)		
Mean number of daily smoked cigarettes	24.63 ± 14.36	29.05 ± 15.86	0.97 [0.94–1.01]	NS
Mean number of visits to the clinic	6.74 ± 3.14	4.48 ± 2.86	1.23 [1.04–1.46]	0.012*
Score of perceived self-confidence to quit			1.7 [0.33–8.54]	NS
≥ 5	17 (25.4)	50 (74.6)		
< 5	2 (16.7)	10 (83.3)		
Score of motivation			0.67 [0.21–2.09]	NS
> 12	13 (21.3)	48 (78.7)		
≤ 12	6 (28.6)	5 (71.4)		
Fagerström score			1.01 [0.36–2.82]	NS
< 7	9 (23.1)	30 (76.9)		
≥ 7	10 (23.3)	33 (76.7)		

*NS* not significant

*p < 0.05

## Discussion

The tobacco use may alter the function of the serotoninergic neurons by the intermediary of at least two of these compounds: nicotine and the bêtacarbolines. This finding reinforces the hypothesis that some psychiatric disorders are probably associated with the consumption of tobacco, because those several compounds of tobacco entail sustainable disturbances in the neurobiological cerebral functioning, including those of the serotonergic system [24]. This study was conducted to assess the effect of psychological problems (depression and anxiety) on the tobacco cessation among smokers attending the smoking cessation clinic of the University Hospital of Sahloul of Sousse.

The existence of an association between anxio-depressive disorders and smoking is clearly established. Depression is the psychiatric comorbidity the better documented. In fact, the prevalence of smoking is higher among patients with major depression. Similarly, the percentage of major depressive disorder was two times higher in smokers versus non-smokers. Thus, the risk of major depressive episode is increased during the 6 months following the smoking cessation [25]. In our study, the prevalence of anxio-depressive disorder was comparable to that found in the study of Fakhfakh et al. in Tunisia (47.2%) [6] using the HAD scale to identify anxio-depressive disorders. It is also comparable to that found by Lasser et al. (55.3%) [26] where rates of smoking and tobacco cessation were studied according to the number and type of psychiatric diagnosis, assessed by a modified version of the Composite International Diagnostic Interview.

Smokers with comorbid depressive disorders are more prone to become dependent on nicotine, to progress to a more severe level of dependence, and to experience more severe nicotine withdrawal symptoms than smoker without depressive disorders [27]. In addition, it has long been recognized that the consumption of tobacco allowed some smokers to control their anxiety as a self-medication. It also seems that smoking cessation can be accompanied by an improvement of the anxiety [25].

Based on the scores of the HAD scale revealed during the first consultation, we have noted 43.4% of anxiety disorder among smokers and 37.6% of depressive disorder with average scores of 10.67 and 6.49 respectively for the anxiety and depression. In the study conducted by Fakhfakh et al. in Tunisia, among 70 patients who responded to the HAD test, they found 22.9% having a depressive disorder and 20% patients with anxiety disorders [6]. The presence of a note of anxiety was significantly higher among women than among men. This difference was not significant in our study, which can be explained by the small size of the sample.

Some research has indicated that smoking helps calm some of the symptoms like restlessness, agitation, and irritability in major depression disorder. Perhaps these smokers are attempting to use smoking as a way to relieve themselves of the symptoms of their depression which could make it extremely difficult for the person to quit smoking. Research has indicated that having this diagnosis does present a challenge for quitting smoking. Among smokers on a Quitline in California, smokers with current major depression were much less likely to have quit for at least 30 days at the time of the 2-month evaluation, compared to those with minimal depressive symptoms [28]. In our study, 55.2% of smokers with anxio-depressive disorders have tried at least once to stop smoking. We did not find a significant difference in the attempts to quit between depressed subjects, anxious, anxio-depressed, and normal subjects. According to the study conducted by Fakhfakh et al. [6], three quarters of patients have made at least one attempt to quit smoking. However, the difference with smokers without anxio-depressive disorder was not significant. According to another study conducted by Mumtaz et al. on 1725 participants [29], the rate of previous attempts to stop was significantly higher among weakly dependent smokers. In our study, the mean score of motivation was 4.36 ± 4.63. This score was similar among both smokers not presenting an anxio-depressive disorder and anxio-depressed smokers.

According to Mendelsohn [30], contrary to popular belief, evidence suggests that smokers with depression are highly motivated to quit smoking. In fact, in a study conducted by Haukkala et al. [31], female smokers who were depressed were more motivated to quit than those with a lower depression score. The mean score of self-confidence to quit was 6.57 ± 2.41 in anxious smokers, 6.33 ± 2.48 in depressed smokers, and 6.14 ± 2.65 in anxio-depressed smokers. On the contrary, in the study of Fakhfakh et al., subjects with a history of depression had significantly a lower mean score of self-confidence to quit [6]. Zvolensky et al. [10], in their survey on more than 3000 subjects, have shown that depression is more common among smokers than non-smokers and that smokers with a history of depression were significantly less likely to quit smoking.

Furthermore, in our study, anxiety and depression were not correlated to the number of cigarettes smoked per day, nor to the average rate of exhaled CO in air. Our findings were in line with those of Zvolensky et al. [10].

The mean Fagerström score was of 6.26 ± 2.38. It was significantly higher among smokers with an anxio-depressive disorder during the first consultation. This score was 7.32 in the study of Fakhfakh and it was also significantly higher among smokers with an anxio-depressive disorder [6].

Strongly dependent smokers with anxio-depressive disorder are frequently encountered in the smoking cessation clinic. Hence, in a study by Largue et al. [32] on 517 smokers, a frequency of 34% of strongly dependent anxio-depressive smokers was found with variable intensities of depression. The association between smoking and major depression is stronger if there is a dependency to nicotine [10]. The results found by Mumtaz et al. [29] confirmed that the symptoms of depression, anxiety, or agoraphobia were more severe in smokers strongly dependent than among non-dependent ones. These conclusions were in line with those of Coutino et al. [33] and Pedersen and Von Soest [34], where heavy smoking and a strong dependence to nicotine were associated with a high rate of depression and anxiety.

Tobacco cessation is a difficult process because of the multiple dependencies (pharmacological, behavioral, and psychological). Among the objectives of our survey was to estimate the impact of the anxio-depressive disorder among smokers of the smoking cessation clinic. We have defined tobacco cessation as a total abstinence for a duration equal to or more than a week. About one third (33.9%) of our participants succeeded in the withdrawal. For the same definition, a study made at the Institute Salah Azzaiez among smokers in their smoking cessation clinic in 2004 [35] found a rate of withdrawal of 27%. Our rate could be explained by the strong dependency found in more than half of the smokers as well as the high number of people dropped out and had been counted as a failure in the analysis. In addition, 74.1% of smokers have benefited of only one or two visits.

The rate of tobacco cessation among smokers with anxio-depressive disorder of our study varied according to the duration maintained of tobacco cessation; it was 51.2% at 1 month which decreased gradually to reach 18.3% at 12 months.

In our study, the follow-up to the consultation was the predictive factor of successful tobacco cessation at 6 months. On the other hand, in multivariate analysis, no factor appeared as a predictive factor of relapse at 1 year. This finding was different from that found in the study by KesKesBoudawara et al. [36] and by Underner et al. [37], where only a follow-up prematurely interrupted was significantly associated with the relapse.

Tobacco use is often considered as a tool to overcome difficult situations caused by social anxiety and lack of confidence in smokers suffering from anxio-depressive disorders. These disorders are more frequent among women, explaining in part their greater difficulty to stop smoking. The other reasons are the greater frequency of depressive disorders, the less effectiveness of nicotine substitutes, and the more important fear to gain weight once stopping smoking among women [38]. Furthermore, smokers would find in the consumption of cigarettes a way to reduce the negative emotions including the negative symptoms of withdrawal [23].

Sonntag et al. [39] have highlighted through a longitudinal study on 3021 adolescents and young adults, an association between social phobia, and anxiety disorders and the occurrence of a nicotinic dependency. The recognition of the importance played by the negative emotions in the smoking behavior has led some authors to suggest the use of antidepressants and anxiolytics as a treatment for tobacco dependence [40]. Furthermore, some smokers who are trying to quit could be helped by concurrent treatment for depression.

### Study limitations

The present study has some limitations with regard to the generalizability of the findings. First, the sample size was small particularly the number of anxio-depressed smokers compared to many other studies. Second, for some variables, we did not have the necessary information which diminished further our sample size. Finally, perhaps going further with the study may increase the sample size.

## Conclusion and recommendations

The association of anxio-depressive disorder with tobacco use is very frequent, since it is found in more than 50% of the strongly dependent smokers attending the smoking cessation clinics. These disorders are more severe as the level of dependency is higher. Our findings did not conclude on the association between anxio-depressive disorder and smoking dependence. However, we found that the rate of anxio-depressive disorder among smokers seeking for tobacco cessation was high among our population study. We recommend to systematically assess the presence of anxio-depressive disorder among smokers attending smoking cessation clinics so we will not miss the presence of such disorder that may delay tobacco cessation.

## References

[1] Cardozo Pawlina MM, Regina de Cássia R, Mariano ME, Clovis B (2015). Depression, anxiety, stress, and motivation over the course of smoking cessation treatment. J Bras Pneumol.

[2] Levy D, de Almeida LM, Szklo A (2012). The Brazil SimSmoke policy simulation model: the effect of strong tobacco control policies on smoking prevalence and smoking-attributable deaths in a middle-income nation. PLoS Med.

[3] WHO. Tobacco. Fact sheet no.339 Updated March 2018.[cited 2018, March 8] Available from: http://www.who.int/mediacentre/factsheets/fs339/en/

[4] Centers for Disease Control and Prevention, National Center for Chronic Disease Prevention and Health Promotion, Office on Smoking and Health (2010). How tobacco smoke causes disease: the biology and behavioral basis for smoking-attributable disease: A Report of the Surgeon General.

[5] Centers for Disease Control Prevention (CDC) (2011). Quitting smoking among adults--United States, 2001-2010. MMWR Morb Mortal Wkly Rep.

[6] Fakhfakh R, Aouina H, Gharbi L, Hsairi H, Achour N, Largue G (2003). Dépendance tabagique et troubles anxio-dépressifs chez le fumeur tunisien. Rev Mal respire.

[7] Reichert J, Araújo AJ, Gonçalves CM, Godoy I, Chatkin JM, Sales MP (2008). Smoking cessation guidelines-2008. J Bras Pneumol.

[8] Piper ME, Cook JW, Schlam TR, Jorenby DE, Baker TM (2011). Anxiety diagnoses in smokers seeking cessation treatment: relations with tobacco dependence, withdrawal, outcome and response to treatment. Addiction.

[9] Martins KC, Seidl EM (2011). Mudança do comportamento de Fumarem participantes de grupos de Tabagismo. Psic Teor Pesq.

[10] Zvolensky MJ, Farris SG, Leventhal AM, Ditre JW, Schmidt NB (2015). Emotional disorders and smoking: relations to quit attempts and cessation strategies among treatment-seeking smokers. Addict Behav.

[11] Grant BF, Hasin DS, Chou SP, Stinson FS, Dawson DA (2004). Nicotine dependence and psychiatric disorders in the United States: results from the National Epidemiologic Survey on Alcohol and Related Conditions. Arch Gen Psychiatry.

[12] Leventhal AM, Ray LA, Rhee SH, Unger JB (2012). Genetic and environmental influences on the association between depressive symptom dimensions and smoking initiation among Chinese adolescent twins. Nicotine Tobacco Res.

[13] Audrain-McGovern J, Rodriguez D, Rodgers K, Cuevas A (2011). Declining alternative reinforcers link depression to young adult smoking. Addiction.

[14] McKenzie M, Olsson CA, Jorm AF, Romaniuk H, Patton GC (2010). Association of adolescent symptoms of depression and anxiety with daily smoking and nicotine dependence in young adulthood: findings from a 10-year longitudinal study. Addiction.

[15] Peasley-Miklus CE, McLeish AC, Schmidt NB, Zvolensky MJ (2012). An examination of smoking outcome expectancies, smoking motives and trait worry in a sample of treatment-seeking smokers. Addict Behav.

[16] Leventhal AM, Japuntich SJ, Piper ME, Jorenby DE, Schlam TR, Baker TB (2012). Isolating the role of psychological dysfunction in smoking cessation: relations of personality and psychopathology to attaining cessation milestones. Nicotine Tob Res.

[17] Weinberger AH, McKee SA (2012). Gender differences in smoking following an implicit mood induction. Nicotine Tob Res.

[18] Zvolensky MJ, Gibson LE, Vujanovic AA, Gregor K, Bernstein A, Kahler C (2008). Impact of posttraumatic stress disorder on early smoking lapse and relapse during a self-guided quit attempt among community-recruited daily smokers. Nicotine Tob Res.

[19] Piper ME, Cook JW, Schlam TR, Jorenby DE, Baker TB (2011). Anxiety diagnoses in smokers seeking cessation treatment: relations withtobacco dependence, withdrawal, outcome and response to treatment. Addiction.

[20] Heatherton TF, Kozlowski LT, Frecker RC, Fagerstrom KO (1991). The Fagerstrom test for nicotine dependence: a revision of the Fagerstrom Tolerance Questionnaire. Br J Addict.

[21] The National Health Service (NHS), London Clinical Senate. Helping smokers quit. The expired carbon monoxide test. [cited 2016 March 12]. Available at: http://www.londonsenate.nhs.uk/wp-content/uploads/2015/04/The-expired-carbon-monoxide-CO-test-guidance-for-health-professionals.pdf

[22] Aubin HJ, Lagrue G, Legeron P, Azoulaï G, Pélisolo S, Humbert R (2004). Questionnaire de motivation à l’arrêt du tabac (Q-MAT). Alcool Addictol.

[23] Zigmond AS, Snaith RP (1983). The hospital anxiety and depression scale. Acta PsychiatrScand.

[24] De Beaurepaire R (2004). Effets du tabac sur les neurones sérotoninergiques. Comprendre la dépendance pour agir. Rapport.

[25] Fakhfakh R, Lagrue G (2002). Dépression, dépendance tabagique et nicotine. Archs Inst Pasteur Tunis.

[26] Lasser K, Boyd JW, Woolhandler S, Himmelstein DU, McCornick D, Bor DH (2000). Smoking and mental illness: a population based prevalence study. JAMA.

[27] Johnson EO, Breslau N (2006). Is the association of smoking and depression a recent phenomenon?. Nicotine Tob Res.

[28] Hebert KK, Cummins SE, Hernández S, Tedeschi GJ, Zhu S (2011). Current major depression among smokers using a state quitline. AJPM.

[29] Mumtaz J, Willem AJ, Pim C, Brenda WJHP (2012). Association of smoking and nicotine 18ependence with severity and course of symptoms in patients with depressive or anxiety disorder. Drug Alcohol Depend.

[30] Mendelsohn C (2012). Smoking and depression: a review. Aust Fam Physician.

[31] Haukkala A, Utela A, Vartiainen E, McAlister A, Knekt P (2000). Depression and smoking cessation: the role of motivation and self-efficacy. Addict Behav.

[32] Largue G, Dupont P, Fakhfakh R (2002). Troubles anxieux et dépressifs dans la dépendance tabagique. L’Encéphale.

[33] Coutino AM, Velasco SR, Icaza M (2009). Association between smoking and minimal-mild depressive symptomatology in heavy smokers. Salud Mental.

[34] Pedersen W, Von Soest T (2009). Smoking, nicotine dependence and mental health among young adults: a 13-year population-based longitudinal study. Addiction.

[35] Ben Ayoub W, Djoufelkit K, Toenbern-Delbarre A, Fakhfakh R, Ben Mansour A, Ben Ayed F (2008). La consultation d’aide au sevrage tabagique de l’Institut de cancérologie Salah Azzaiez de Tunis. RevEpidemiol Santé Publique.

[36] KeskesBoudawara N, Bousoffara L, Touil I, El Fahem N, Sakka M, Knani J (2013). Efficacité et facteurs prédictifs d’échec d’une consultation hospitalière d’aide au sevrage tabagique. Rev Ma. Respir.

[37] Underner M, Ingrand P, Favreau M, Mura P, Meurice JC (2004). Intérêt des principaux indicateurs du tabagisme lors de la première consultation de sevrage tabagique. Rev Mal Respir.

[38] Dupont P, Largue G (2002). L’arrêt du tabac est-il plus difficile chez les femmes ?. Médecine au féminin.

[39] Sonntag H, Wittchen HU, Hofler M, Kessler RC, Stein MB (2000). Are social fears and DSM-IV social anxiety disorders associated with smoking and nicotine dependence in adolescents and young adults. Eur Psychiatry.

[40] Hughes JR, Stead LF, Lancaster T (2007). Antidepressants for smoking cessation. Cochrane Database Syst Rev.

